# Monitoring Training Adaptation and Recovery Status in Athletes Using Heart Rate Variability via Mobile Devices: A Narrative Review

**DOI:** 10.3390/s26010003

**Published:** 2025-12-19

**Authors:** Michael R. Esco, Andrew D. Fields, Matthew A. Mohammadnabi, Brian M. Kliszczewicz

**Affiliations:** 1 Department of Kinesiology, The University of Alabama, 620 Judy Bonner Drive, Tuscaloosa, AL 35401, USA; 2 Department of Exercise Science and Sports Management, Kennesaw State University, 1000 Chastain Road NW, Kennesaw, GA 30144, USA

**Keywords:** HRV, autonomic modulation, wearable device, athlete performance, sport science

## Abstract

Heart rate variability (HRV) is a non-invasive biomarker that reflects autonomic nervous system dynamics, providing valuable insights into physiological adaptation, stress, and recovery in athletes. Among the various HRV metrics, the root mean square of successive differences (RMSSD) has emerged as a robust and practical measure due to its strong association with parasympathetic activity, ease of calculation, and reliability in both short- and ultra-short-term recordings. This review examines the methodological considerations for using HRV to monitor training adaptations and recovery status in athletic populations. We highlight the superiority of routine, near-daily HRV measurements over isolated assessments, emphasizing the utility of weekly averages and the coefficient of variation (CV) to capture both chronic adaptations and acute homeostatic perturbations. Additionally, we discuss the selection of HRV devices, data recording procedures, and strategies to enhance athlete compliance. While RMSSD offers significant advantages for field-based monitoring, we also address its limitations, including its sole focus on parasympathetic activity and susceptibility to external confounders. Future directions include the integration of HRV data with other physiological markers and machine learning algorithms to optimize individualized training and recovery strategies. This review provides sport scientists and practitioners with evidence-based recommendations to enhance the application of HRV in both research and real-world athletic settings.

## 1. Introduction

Heart rate variability (HRV) refers to the fluctuation in the time intervals between consecutive heartbeats, reflecting the dynamic interplay between the sympathetic and parasympathetic branches of the autonomic nervous system [1,2]. These fluctuations provide a non-invasive view into autonomic regulation, offering insights into physiological recovery, stress, and overall health. Accordingly, HRV has emerged as a foundational biomarker of cardiac autonomic modulation in both clinical and athletic contexts [2,3].

From a practical perspective, HRV serves as an objective physiological indicator that relates to how well the body is coping with physical stress. When measured at rest, high HRV indicates a predominance of parasympathetic activity, suggesting a relaxed and recovered state [4]. Conversely, low HRV reflects greater sympathetic activation or reduced vagal tone, often associated with stress or fatigue [4]. However, HRV responses during prolonged or highly demanding activities can be more complex. While most work demonstrates a blunted HRV response under substantial physiological strain [5], other studies in military, tactical, and overreached athletic populations have reported paradoxical increases in HRV despite significant stress [6,7,8]. These findings emphasize that HRV should be interpreted within the broader training context and longitudinal trends, instead of isolated recordings.

In sports contexts, a common approach to using HRV is through HRV-guided training, which involves making adjustments to a training session’s intensity based on waking HRV values [9,10]. While this model offers a practical method for day-to-day exercise prescription, HRV can also be used to evaluate broader physiological processes related to training adaptation and recovery [11,12,13,14,15]. Adaptation refers to the long-term autonomic and physiological adjustments that occur in response to repeated training stimuli [11], which are often reflected in gradual shits in weekly HRV trends [3]. In contrast, recovery relates to the short-term physiological response following physical stress and the body’s ability to restore homeostasis [12,14], which can be monitored through acute changes in HRV relative to an athlete’s individual baseline [3]. Recovery and adaptation are interdependent processes, as adequate recovery supports favorable adaptation, whereas impaired recovery may signal or contribute to maladaptation [13]. Thus, tracking HRV trends, particularly through weekly mean values and the coefficient of variation (CV), provides insight into both adaptation and recovery [3]. Rather than guiding single-day adjustments, these longitudinal patterns help monitor overall training status across weeks or training blocks and support decisions related to load management, performance readiness, and recovery strategies [14,15].

Over the past few decades, a variety of HRV metrics and devices have emerged, enabling the monitoring of recovery and long-term adaptation in a variety of settings [16]. While this diversity may prove advantageous to consumers and researchers, it also highlights potential pitfalls, particularly if selected procedures do not align with specified goals. Given this methodological heterogeneity, clearer guidance is needed regarding the appropriate use of mobile HRV tools. However, existing reviews on this topic have generally focused on laboratory-based HRV methods or broad conceptual overviews, with limited attention to the unique methodological challenges associated with mobile platforms. Currently, there is no single resource that integrates device-specific considerations, ultra-short and weekly HRV metrics, and practical issues related to data interpretation and athlete compliance. Thus, a clear, integrated synthesis that consolidates device-specific considerations, ultra-short and weekly HRV metrics, and practical issues related to real-world data interpretation is still lacking. The aim of this narrative review is to address key methodological considerations for using mobile HRV devices in athlete monitoring and to provide practical recommendations for optimizing their application in assessing training adaptation and recovery. Topics discussed include the selection of HRV metrics and mobile devices, best practices for recording procedures, and strategies for interpreting HRV data in applied sport settings.

## 2. Methods

This review was conducted as a narrative synthesis of conceptually relevant evidence to provide practical guidance for applied athlete monitoring via wearable HRV sensors and mobile technologies. The aim was to contextualize emerging practices, highlight methodological considerations, and connect empirical findings to real-world application rather than exhaustively summarize all available studies. The authors’ intentions were to align the review with established recommendations outlining when narrative reviews are appropriate within scientific scholarship [17,18,19].

Literature searches were conducted between January and August 2024 using the databases and example search terms summarized in Table 1. Searches were further supplemented by manual screening of reference lists from key empirical papers and HRV-focused reviews. No publication date limits applied to the search criteria.

Study selection was guided by methodological rigor, ecological relevance to athlete monitoring, and applicability of HRV assessment using mobile devices. Evidence was synthesized qualitatively by prioritizing peer-reviewed studies that: evaluated HRV under resting conditions and in response to acute exercise; explored longitudinal HRV responses to training; validated ultra-short-term recordings and wearable devices; addressed methodological considerations relevant to field-based monitoring; and had direct relevance to using HRV in applied sport settings. Studies that focused primarily on areas outside of the main purpose of this review, such as in the realms of clinical disorders or pharmacological interventions, were generally excluded unless they offered essential mechanistic insight.

Because this was a narrative review, predefined inclusion/exclusion criteria and formal risk-of-bias scoring were not applied. The authors acknowledge the potential for selection bias inherent to narrative syntheses [17]. However, every effort was made to reflect a balanced cross-section of the most influential and methodologically sound work related to the use of mobile HRV devices and recording procedures for athletic contexts.

## 3. Choice of HRV Metric

### 3.1. The Common Parameters

HRV analysis begins with the detection of R-R intervals, also referred to as inter-beat intervals, which represent the time between successive R-waves in the QRS complex of an electrocardiogram (ECG). While modern wearable technologies now allow for the recording of these intervals using validated mobile devices (discussed later in this paper), ECG remains the gold standard due to its superior temporal resolution and ability to precisely detect the fiducial point of each R-wave [2]. Once extracted, these R-R intervals form the basis for calculating the various HRV metrics, which are typically categorized into three primary domains: time-domain, frequency-domain, and non-linear measures. Table 2 provides an overview of the most widely used HRV indices, outlining their physiological significance and implications for athlete monitoring.

The selection of the appropriate HRV metric depends on the context of measurement and the goals of research or practice [1,2]. Time-domain metrics are often preferred in mobile settings due to their ease of computation and interpretation [3,20]. Frequency-domain metrics may offer richer insight into autonomic balance, especially when assessed under tightly controlled laboratory conditions [2,21,22,23]. However, controversy exists concerning the physiological underpinning of the low-frequency domain, and the complexity and respiratory sensitivity of these metrics can limit field-based applications [23,24,25]. Non-linear HRV metrics evaluate the complexity and unpredictability of heart rate patterns, which can be advantageous during non-steady state assessments [26,27,28,29]. Notably, the Poincaré plot-derived SD1 and the time-domain metric RMSSD are mathematically equivalent and, hence, both reflect vagally mediated HRV [30]. In addition, because most HRV variables follow a non-normal, right-skewed distribution, it is standard practice to apply a natural logarithmic transformation (e.g., lnRMSSD) before statistical analysis to stabilize variance and meet assumptions for parametric testing [20].

### 3.2. RMSSD as the Standard for Athletic Monitoring

Among the available HRV metrics, RMSSD is arguably the most common for field-based monitoring [3]. Calculated as the root mean square of the successive R-R interval differences, RMSSD primarily reflects parasympathetic (vagal) activity [31,32,33]. Numerous characteristics position RMSSD as the preferred HRV metric among athletes [32,33,34,35,36,37,38]. For instance, it is easier to compute and interpret than frequency-domain and non-linear metrics [1,4]. Furthermore, it remains relatively stable across a range of spontaneous breathing rates [39,40]. It reflects parasympathetic activity at rest and in response to acute exercise [41]. In addition, RMSSD demonstrates superior reliability and is less sensitive to external factors compared to frequency-domain metrics [42,43,44]. Therefore, the need for strict control during data collection is minimized with RMSSD.

Perhaps its most advantageous characteristic related to athlete monitoring in mobile or field-based settings is the utility of RMSSD under ultra-short timeframes. Traditional standards for short-term HRV assessment recommend a 5 min stabilization period followed by a 5 min recording period [2], a protocol often considered impractical in dynamic sport environments. However, because RMSSD is less susceptible to respiratory variations and non-physiological influences on signal drift that often confound other HRV indices [1,45], it appears to be suitable when assessed under ultra-shortened recording durations.

Indeed, several studies have shown that RMSSD maintains strong agreement with standard recording durations (i.e., 5 min stabilization preceding a 5 min recording) when derived from a 1 min segment that follows a 1 min stabilization period [31,46], and that this reliability holds irrespective of body position [47]. Similarly, Munoz et al. [48] compared multiple HRV indices across durations ranging from 10 s to 5 min and found RMSSD exhibited the strongest correlation (r > 0.90) with standard-length recordings, even at durations as short as 30 s. Further research has demonstrated that 1 min RMSSD recordings among athletes produced values nearly identical to conventional 5 min recordings across acute bouts of exercise [31] and long-term training periods [49].

### 3.3. Section Summary

Based on the collective evidence, RMSSD can be recommended as the primary HRV metric for field-based monitoring in athletes. Its strong association with parasympathetic activity and minimal sensitivity to respiratory fluctuations make it an ideal choice for tracking physiological trends over time. Additionally, RMSSD’s ease of calculation and its accuracy in ultra-short-term recordings across various body positions and training conditions enhances its practicality in real-world athletic settings. Therefore, this metric appears to be an asset for athletes, coaches, and practitioners seeking valid physiological feedback with minimal disruption to training or competition routines. However, it should be noted that RMSSD is not without limitations, and several practical and physiological considerations relevant to its interpretation are outlined in Section 7.

## 4. Choice of Mobile HRV Device

As previously mentioned, HRV is most accurately measured by collecting heart rate data via ECG and calculating it using specialized acquisition software. However, this approach is practically limited, requiring the need for laboratory equipment, specialized expertise, and controlled testing conditions, making it inconvenient for routine HRV monitoring in field settings. Although mobile-based HRV devices have gained popularity due to their convenience and accessibility, a key concern relates to their accuracy relative to ECG.

### 4.1. Accuracy of Various Mobile HRV Devices

A meta-analysis by Dobbs et al. [16] compared HRV data from 23 studies using portable sensors, including chest straps, wrist-worn monitors, and smartphone-based photoplethysmography (PPG) applications, and found small but acceptable discrepancies relative to ECG for all devices. Among the available HRV metrics, RMSSD consistently demonstrated the lowest error across devices, regardless of sensor type or body position [16]. Subsequent validation studies have similarly shown strong agreement between ECG and mobile HRV devices, including smartphone cameras [50,51,52], wristwatches [53,54], rings [55], chest straps [56], and ambulatory blood pressure devices [57]. Accuracy may also depend on recording duration, as a one-minute measurement is generally sufficient for RMSSD [16], whereas frequency-domain parameters typically require at least three-to-five minutes [51].

Furthermore, PPG-based devices perform best under resting conditions but lose accuracy during or immediately after exercise because of motion artefacts and vascular changes [58,59,60]. This is likely due to PPG and ECG being physiologically distinct signals. PPG reflects peripheral pulse dynamics rather than cardiac electrical activity [61]. Recent work suggests that these fundamental differences may introduce systematic variability between modalities, particularly during exercise, when vascular and pulse transit time changes can distort PPG signals relative to ECG [61].

Therefore, mobile devices appear to be suitable for measuring resting HRV, particularly for ultra-short RMSSD. However, their use during or immediately following exercise should be approached with caution due to the potential for reduced signal accuracy. Nevertheless, when applied under appropriate conditions, mobile HRV devices provide a practical and valid means of monitoring autonomic trends in athletic settings.

### 4.2. Real-World Practicality of Different Mobile HRV Devices

Beyond accuracy, practical considerations such as comfort, convenience, and cost may influence device selection. Wrist-worn and ring-based devices allow continuous data collection and are particularly effective for nocturnal recordings [54,62,63,64]. Many also provide additional health-related metrics such as heart rate, sleep, and oxygen saturation. Chest strap monitors yield highly accurate ECG-like signals [65] but require proper placement/contact and removal, which some users may find inconvenient and not practical for long-term continuous recordings. Wrist, ring, and chest-worn devices also tend to be more expensive than smartphone-based alternatives. In contrast, smartphone applications that rely on PPG, either through a connected finger sensor [66] or the phone’s camera [58,67], are inexpensive, portable, and ideal for consistent morning recordings [68,69]. These applications may also enhance athlete compliance because they are low-cost, easy to use, and often integrate wellness questionnaires, providing a practical solution for tracking recovery and adaptation through daily HRV measures. Ultimately, the optimal device depends on the athlete’s needs, monitoring frequency, and preferred measurement context. Table 3 summarizes commonly used mobile HRV devices, including example manufacturers, advantages, limitations, and validation findings.

It is also important to note that many commercially available HRV mobile devices (e.g., smartphone applications, wearables, etc.) automatically apply a log transformation to RMSSD. However, because lnRMSSD values are not intuitively interpretable by consumers, several systems multiply the transformed value by a constant (such as 20) to rescale the output to a more user-friendly format [30,66]. For example, a raw lnRMSSD value of 4.2 might be presented as an HRV “score” of 84. While these adjusted, arbitrary unit values are useful for tracking trends, practitioners and consumers should understand how such scores are derived to ensure consistency when interpreting HRV data across different platforms. In addition, because transformed scores do not retain the original time-domain scale, they may not be directly comparable to raw RMSSD values in milliseconds.

### 4.3. Section Summary

From a practitioner’s perspective, the cost–benefit of using portable devices for HRV monitoring often takes precedence over minor errors in accuracy. Furthermore, the level of error across portable devices, including wrist-worn monitors, chest straps, and smartphone-based applications, does not significantly differ when evaluating RMSSD [16]. As such, meaningful interpretation of longitudinal HRV trends depends largely on the consistency and frequency of measurement [67,68,69].

## 5. HRV Recording Procedures

### 5.1. The Importance of Daily or Near-Daily HRV Measures

Traditionally, HRV has been assessed using isolated, single-time-point measures, particularly in clinical settings [70]. However, this approach has several limitations. Single-time-point HRV measures are highly susceptible to transient fluctuations caused by daily stressors, disruptions in sleep, environmental factors, and measurement inconsistencies, making them less reliable for tracking meaningful physiological changes [38]. Additionally, isolated recordings do not account for baseline HRV, which requires frequent measurements over at least a week to establish [3]. Without this baseline, it is difficult to interpret whether a given HRV value reflects an individual’s homeostatic state or a temporary deviation. Nevertheless, because inter-individual differences in HRV can be influenced by factors unrelated to fitness or health [71], a lower HRV compared to a peer does not necessarily indicate poorer physiological status. Therefore, the focus in athletic monitoring should be on intra-individual changes, with frequent HRV assessments taken over time within an individual rather than single measurement comparisons pre-to-post training or relative to others. Therefore, establishing personalized interpretation of long-term HRV trends is essential for optimizing training and recovery strategies.

To accomplish this, athletes and practitioners should aim for daily HRV recordings over a full 7-day period and monitor trends on a week-to-week basis (see Section 6, for more detail). However, emerging evidence suggests that fewer measurements per week may still be sufficient to capture meaningful weekly trends. Plews et al. [72] investigated the minimum number of morning HRV recordings needed to approximate a full week’s average in both recreationally trained and highly trained triathletes. Their findings indicated that a minimum of five recordings per week was necessary for recreationally trained athletes, whereas recordings over just three days were sufficient for highly trained individuals to achieve acceptable agreement with the 7-day reference [72]. Accordingly, collecting 3 to 5 well-controlled, technically consistent morning recordings may provide a sufficiently accurate weekly HRV profile when daily monitoring is not feasible. Nevertheless, to ensure the highest validity and reliability of weekly HRV metrics, daily measurements with minimal missed days remain the recommended standard.

### 5.2. Timing Considerations for Field-Based HRV Recordings

A critical element of accurate HRV assessment is consistency in the timing of data collection. Nocturnal HRV recordings obtained during deep sleep are considered highly reflective of basal parasympathetic tone [73]. Additionally, this approach limits the influence of environmental and behavioral factors that can confound waking HRV assessments [74]. However, existing challenges limit the widespread utility of nocturnal HRV monitoring in athletes. While several commercially available wearables now offer automated nocturnal HRV tracking with reasonable accuracy [54,62,75], the practical feasibility remains limited due to costs, variability in measurement quality, and difficulties extrapolating and interpreting the data. Although nocturnal recordings represent a promising future direction for athlete monitoring, standardized, waking HRV assessments remain the most practical and scientifically supported method for high-frequency, field-based monitoring at present.

Therefore, it is recommended to record HRV as close to awakening as possible. Supporting this approach, Mishica et al. [76] demonstrated no significant differences and strong correlations between nocturnal and morning RMSSD when measured across several weeks in endurance athletes. Additionally, Williams et al. [77] has shown that HRV measures upon waking were sensitive to changes in resistance training loads across different microcycles, whereas measurements taken later in the day were less informative. Furthermore, Sherman et al. [78] suggested that morning RMSSD recordings were more strongly associated with performance in 2000 m rowing in competitive rowers when compared to recordings taken later in the day. Findings such as these are likely due to transient fluctuations in autonomic nervous system activity that can mask the relationship between HRV and physical readiness. For instance, bladder or bowel distension and digestive activity modulate vagal tone, whereas caffeine consumption stimulates sympathetic activity [79]. To minimize these influences, athletes should record HRV soon after waking and following elimination, but before consuming food or stimulants.

### 5.3. Body Positioning for Field-Based HRV Recordings

Another important consideration is the body position during HRV recording. The supine position is often utilized in research studies due reduced sympathetic activation [44]. However, it may not be practical during morning self-assessments, as individuals might inadvertently fall back asleep [3]. Moreover, elite endurance athletes often exhibit training-induced bradycardia and may experience “parasympathetic saturation” when measured in the supine position [38]. This phenomenon may be a result of continuously elevated levels of acetylcholine within the cardiac nodal synapses, resulting in excessively low resting heart rates and deceptively low HRV values [80]. Therefore, HRV should preferably be recorded in the seated position, as this posture yields values more reflective of training adaptations than those obtained in the supine position [81]. However, for athletes with an excessively low resting heart rate (e.g., <40 beats·min^−1^), the standing position may be more appropriate to avoid parasympathetic saturation [38,82]. Regardless of the chosen posture, maintaining the same body position for every recording is crucial, in that HRV values differ significantly across supine, seated, and standing positions and are therefore not interchangeable [83]. In order to ensure reliable longitudinal monitoring, practitioners and athletes should consistently use the same body position during each recording.

### 5.4. Ultra-Short Time Periods for Field-Based HRV Recording

One of the practical limitations of daily HRV monitoring is the time and compliance burden associated with standard 5 min recordings. To address this, the use of ultra-short-term HRV recordings has recently emerged as a practical and scientifically supported approach for daily athlete monitoring. As described in Section 3.2, RMSSD can be derived from brief (1 min) recordings, making this method well suited for routine morning assessments. Based on the findings of a number of studies [31,48,49,84], an optimal protocol involves a 1 min stabilization period, followed by a 1 min recording period using a device that automatically calculates the HRV metric, such as RMSSD or adjusted lnRMSSD. This approach minimizes participant burden while still producing reliable HRV values suitable for trend analysis. Critically, both the stabilization and recording periods should be free of movement, speech, and environmental distractions, and should be conducted under consistent daily conditions when possible.

### 5.5. Section Summary

**Figure 1:**
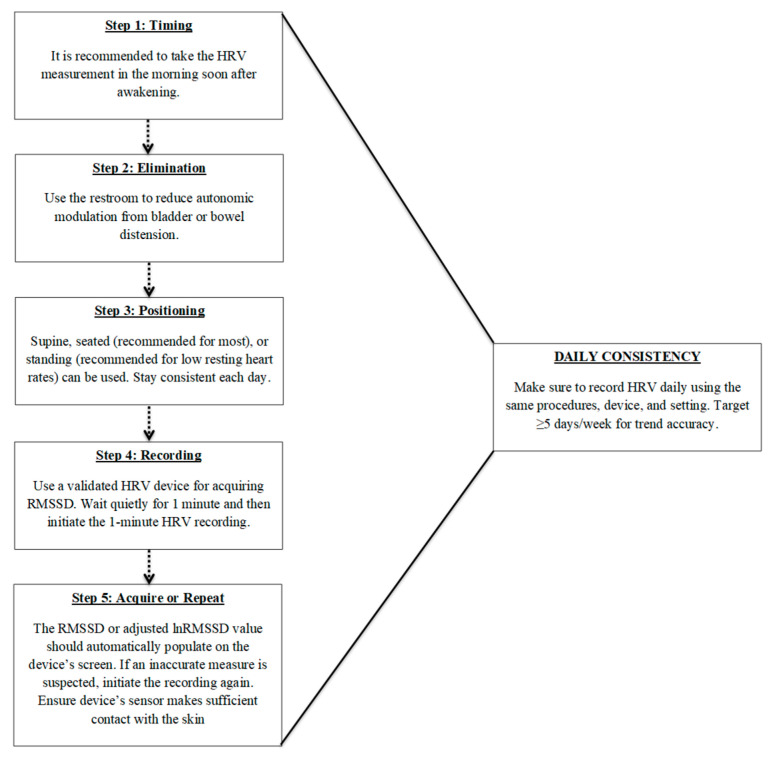
Recommended procedures for daily ultra-short heart rate variability (HRV) monitoring using RMSSD. Steps include: (1) measuring shortly after awakening, (2) voiding the bladder or bowels to reduce autonomic confounding, (3) maintaining a consistent body position (seated preferred), (4) using a validated device for a 1 min recording, and (5) verifying or repeating the measure if signal quality is poor. Daily consistency in procedures and frequency (≥5 days/week) is critical for trend accuracy and meaningful interpretation of RMSSD values.

Accurate and meaningful HRV monitoring requires standardized and consistent recording procedures. Single-time-point measures are limited in their ability to capture true physiological trends, highlighting the importance of frequent, longitudinal assessments that emphasize intra-individual changes rather than comparisons to normative data. Key factors include collecting data at the same time each day, consistent measurement durations, ideally upon waking, and prior to external influences such as food, caffeine, or physical activity. Additionally, maintaining a consistent body position across recordings is critical, with the seated posture often being the most practical and physiologically appropriate for most athletes. Furthermore, ultra-short-term recordings, particularly those using RMSSD, offer a convenient and reliable method for daily monitoring, but should be used consistently for long-term monitoring. To assist with implementation, Figure 1 provides a recommended, step-by-step flowchart summarizing the standardized procedures for ultra-short-term HRV recording. When implemented with consistency, this non-obtrusive protocol enables high-frequency HRV monitoring in field settings, enhancing its feasibility for integration into real-world athlete readiness assessments. This approach has also been successfully utilized in a variety of research designs [85,86,87].

## 6. Data Interpretation

### 6.1. Establishment of a Baseline

As mentioned in Section 5, monitoring intra-individual trends over time is central to effective HRV interpretation [69,86]. To do this appropriately, athletes must first establish a stable personal baseline. In practice, this involves collecting daily waking HRV for at least 7 consecutive days under consistent conditions (e.g., same time of day, posture, and pre-measurement routine) [5,6,20]. The baseline week should be completed during a period of typical training, as excessive load can produce large day-to-day fluctuations and obscure homeostasis [86,87,88]. Once established, this baseline provides the reference point against which future deviations (whether acute or chronic) can be interpreted for load management, readiness, and recovery assessment [69,86]. Because autonomic status evolves with training and adaptation, baseline values may shift over time [69,86,87,88]. Therefore, periodic recalibration may be necessary to ensure that interpretation reflects the athlete’s current physiological state [69,86]. For instance, in applied settings, it may be useful to re-establish a fresh baseline at the beginning of each new training cycle, such as the start of the off-season after athletes have transitioned and recovered from the previous competitive season [86,87,88].

### 6.2. Weekly Mean of HRV

The importance of frequent (e.g., daily) HRV assessment was initially demonstrated by the seminal work of Plews et al. [88]. The study compared the relationship between isolated, single-day vs. weekly averaged RMSSD (RMSSD_MEAN_) values and changes in running performance, as assessed by maximal aerobic speed and timed 10 km running following 9 weeks of training [88]. The findings showed that the changes in maximal aerobic speed and 10 km performance displayed significantly stronger correlations with the changes in RMSSD_MEAN_ values (r = 0.72 and −0.76, respectively) compared to the isolated RMSSD recordings (r = −0.06 and −0.17, respectively).

These findings were further supported by a study involving female collegiate soccer players participating in a 12-week conditioning program [87]. The results showed that the change in RMSSD_MEAN_ from Week 1 and Week 3 was strongly correlated with the prospective change in maximal oxygen consumption at the end of the 12-week training program (r = 0.90). Specifically, players who showed an increase, no change, or decrease in RMSSD_MEAN_ over the first three weeks exhibited a corresponding increase, no change, or decrease in maximal oxygen consumption at Week 12. Additional research suggests that RMSSD_MEAN_ appears to increase the longer an athlete engages in training, which appears more pronounced in those with greater competitive experience [38,89], findings that are likely due to training-induced enhancements in cardiac efficiency [90]. These studies, along with others [91,92], support the use of weekly averaged HRV (specifically, RMSSD_MEAN_) to serve as an internal, objective indicator of the long-term physiological adaptations that underpin changes in physical fitness in response to training.

Typically derived from RMSSD, the weekly mean value is calculated by averaging daily HRV values across a fixed week (e.g., from Sunday to Saturday). This can be mathematically expressed as:

(1) $$Weekly\;Average\;HRV=\frac{Sum\;of\;HRV\;over\;n\;days}{n}\;n\;=\;\mathrm{the}\;\mathrm{number}\;\mathrm{of}\;\mathrm{days}\;\mathrm{during}\;a\;\mathrm{fixed}\;\mathrm{week}\;\mathrm{in}\;\mathrm{which}\;\mathrm{HRV}\;\mathrm{was}\;\mathrm{recorded}$$

Interpreting changes in RMSSD_MEAN_ values depends on the direction of the trend. Increasing trends in the mean value over the long term typically reflect positive training adaptations, improved cardiovascular fitness, and effective exercise prescription strategies over the long term [38,85]. Conversely, declining trends correspond to decreases in physical fitness and may indicate accumulated fatigue, nonfunctional overreaching, or overtraining [38,93,94]. Therefore, shifts in weekly mean HRV can signal whether the training program is effective or if adjustments are needed, such as modifying the training load or encouraging an extended recovery period [38,85].

### 6.3. Weekly Coefficient of Variation in HRV

While the weekly mean HRV provides important insight into longer-term adaptations, it does not account for the day-to-day fluctuations that may occur in response to immediate challenges to homeostasis, which is important for monitoring acute recovery status. This can be captured by calculating the CV of HRV within the same fixed week (e.g., Sunday → Saturday) that the mean value is calculated. The CV offers objective insight into internal, physiological responses and homeostatic perturbations over a short-term period, such as through a week of training [85]. The CV expresses the magnitude of day-to-day fluctuations relative to the weekly mean and hence, is calculated as:

(2) $$HRV\;CV\%=\left(\frac{Standard\;Deviation\;of\;Weekly\;HRV}{Mean\;of\;Weekly\;HRV}\right)\times 100$$

Similar to the weekly mean values, existing research has primarily focused on the CV of RMSSD (RMSSD_CV_). Higher RMSSD_CV_ values purportedly indicate greater perturbations in autonomic homeostasis, often signaling insufficient recovery during a given week [69,85,95]. Conversely, lower values may suggest more stable physiological resilience and a greater readiness to perform [69,85,95]. For example, in a case study by Flatt and Esco [96], a collegiate male cross-country athlete’s weekly RMSSD_CV_ demonstrated a strong correlation (r ≈ 0.92) with race performance over the season. Weeks in which RMSSD_CV_ was lower corresponded to faster completion times, whereas the weeks with higher RMSSD_C_v corresponded to slower race times [96].

### 6.4. Interpreting Both Weekly Metrics

As noted above, RMSSD_MEAN_ and RMSSD_CV_ are calculated from daily RMSSD values recorded throughout a given week. To illustrate this, Table 4 presents an example of 7 consecutive days of arbitrary RMSSD data, along with the corresponding weekly RMSSD_MEAN_ and RMSSD_CV_. For additional context, the standard deviation for the week is also provided, since it is used with RMSSD_MEAN_ to calculate RMSSD_CV_.

In general, RMSSD_MEAN_ and RMSSD_CV_ are moderately-to-strongly inversely correlated, with reported coefficients of approximately *r* ≈ −0.50 [71]. This indicates that increases in one metric are often accompanied by decreases in the other [71]. For instance, favorable adaptations to training are often characterized by concurrent increases in RMSSD_MEAN_ and decreases in RMSSD_CV_, reflecting enhanced autonomic stability and optimized performance [8,36,91,97]. As highlighted by Nakamura et al. [71], training-induced improvements in vagally mediated HRV (i.e., increased RMSSD_MEAN_) may correspond to enhanced homeostatic resiliency (i.e., lowered RMSSD_CV_) and superior responses to elevated training loads. Accordingly, training strategies aimed at increasing RMSSD_MEAN_ and decreasing RMSSD_CV_ may facilitate physiological adaptation, reduce the risk of accumulated fatigue, and enhance an athlete’s capacity to tolerate and recover from intensified training [71,98].

However, despite the inverse relationship between the two metrics being considered moderate-to-strong, it is far from perfect, as each variable reflects a distinct aspect of autonomic function and training status. Therefore, RMSSD_MEAN_ and RMSSD_CV_ should be interpreted independently, particularly since one can change while the other remains stable. This is supported by previous research demonstrating that acute periods of intensified training or short-term accumulated fatigue often correspond with elevated RMSSD_CV_, even though RMSSD_MEAN_ may remain unchanged [86] or slightly decrease [99]. For instance, Flatt and Howells [100] showed no differences in RMSSD_MEAN_ across a 3-week period of intensified training in rugby sevens players, while RMSSD_CV_ slightly increased during the first week but moderately decreased by the third week. In addition, Flatt and Esco [37] demonstrated a decrease RMSSD_CV_ (from ~7% to 4%) following a drop in training load from one week to the next, without a simultaneous change in RMSSD_MEAN_. Furthermore, RMSSD_CV_ was more sensitive to acute adjustments in weekly training load during an 18-week periodized resistance training program in a collegiate hockey player compared to RMSSD_MEAN_ [87]. As such, temporary increases in RMSSD_CV_ may occur during brief periods of overload training as an “alarm” response required for general adaptation [101]. These values may subsequently decrease to reflect positive physiological adaptation to the training stimulus or after a planned deload week intended for recovery [69,85,87].

In contrast, if elevated RMSSD_CV_ values persist for extended periods, such as beyond the duration of an overload microcycle or during typical recovery timeframes, there is a high likelihood that RMSSD_MEAN_ will begin to decrease. This response has been shown in a non-functionally overreached triathlete whose persistent elevated RMSSD_CV_ led to decrements in rolling averaged values [20]. Eventually, both metrics declined, coinciding with poor competition performance and the reactivation of a dormant shingles virus [20]. These observations suggest that persistence elevated RMSSD_CV_ over several weeks may serve as an early warning sign of maladaptation, preceding declines in overall parasympathetic activity (i.e., decreased RMSSD_MEAN_), reduced recovery capacity, and increased risk of illness or injury.

### 6.5. Practical Examples of the Application of RMSSD_MEAN_ and RMSSD_CV_

**Figure 2:**
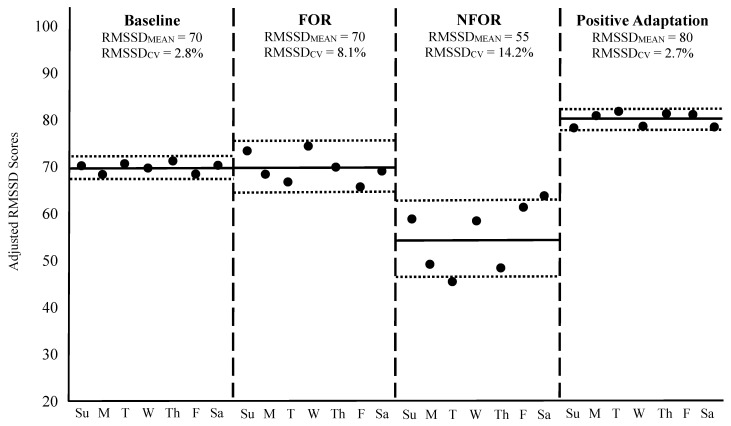
Weekly HRV responses across four different scenarios. The solid black dots represent each day’s RMSSD (log-transformed and adjusted) value. The horizontal solid black lines during each week indicate the RMSSD_MEAN_, while the outside dotted horizontal lines correspond to RMSSD_CV_. The vertical dashed lines separate each week. Su = Sunday, M = Monday, T = Tuesday, W = Wednesday, Th = Thursday, F = Friday, Sa = Saturday, FOR = Functional overreaching, NFOR = Non-functional overreaching.

To contextualize these principles, Figure 2 illustrates four weekly HRV scenarios in a hypothetical athlete, including: (1) a baseline week of typical training, (2) a week of functional overreaching (FOR), (3) a week of non-functional overreaching (NFOR), and (4) a post-training week reflecting positive adaptation to a long-term program. HRV was recorded daily each morning using a validated mobile device that provided an adjusted, log-transformed RMSSD score in arbitrary units. Please note: FOR refers to a short-term performance decrement due to a brief period of intensified training, leading to subsequent performance improvement after adequate recovery [102]. In contrast, NFOR refers to a longer-term period of excessive training loads and is often accompanied by fatigue, mood disturbance, or physiological dysregulation [103,104]. While FOR is an intentional training strategy designed to boast performance, NFOR is unintentional and may lead to the onset of overtraining syndrome [103,104].

During the baseline week shown in Figure 2, RMSSD_MEAN_ was 70 with an RMSSD_CV_ of 2.8%, reflecting a stable physiological state in response to a normal training load. In the FOR scenario, RMSSD_MEAN_ remained unchanged, but RMSSD_CV_ increased to 8.1%, indicative of an acute perturbation to homeostasis often observed during intensified, yet intended, training microcycles [85,87,88]. It is worth noting, however, that a slight decrease in RMSSD_MEAN_ in response to a weekly increase in training load, such as during an FOR microcycle, is not uncommon and may still reflect an appropriate and manageable training stimulus [6,86,92]. In such cases, both the elevated RMSSD_CV_ and any drop in RMSSD_MEAN_ are typically transient and should return to baseline as the athlete adapts to the training stimulus or following a well-timed week of recovery [88].

In contrast, the NFOR week demonstrated both a decline in RMSSD_MEAN_ to 55 and a further elevation in RMSSD_CV_ to 14.2%, suggesting the training load exceeded the athlete’s adaptive capacity for a prolonged period, resulting in insufficient recovery and accumulated fatigue [20,92]. Such changes may necessitate longer recovery or deload periods for the weekly metrics to normalize, to prevent the onset of overtraining syndrome [3,103,104]. However, if these responses remain prolonged, and the athlete reaches a state of chronic overtraining, then RMSSD_CV_ may remain suppressed, but RMSSD_MEAN_ may paradoxically decrease [20]. This decline in day-to-day variability may occur when the autonomic system becomes less responsive due to chronic stress, exhaustion, or the presence of an acute illness, leading to reduced RMSSD_CV_ despite ongoing dysfunction [20]. It is important to note that during periods of FOR or NFOR, athletes may or may not present physical or mental symptoms of fatigue at rest, making the use of RMSSD_MEAN_ and RMSSD_CV_ more urgent when seeking to avoid OR.

Finally, the week representing positive adaptation showed an elevated RMSSD_MEAN_ of 80 and an RMSSD_CV_ similar to baseline (2.7%), signifying improved physiological function, enhanced cardiorespiratory fitness, and successful long-term training adaptation [85]. Although RMSSD_CV_ remained unchanged from baseline, it is important to consider that the baseline value was already quite low. As previously noted, a reduction in RMSSD_CV_, particularly when baseline values are moderately elevated, is also an indicator of positive adaptation. In such cases, lower day-to-day variability reflects greater physiological stability and resilience, supporting the athlete’s improved readiness and capacity to tolerate intensified training loads [71].

For additional examples of interpretation, Table 5 provides a range of weekly RMSSD_MEAN_ and RMSSD_CV_ responses under a variety of real-world training and recovery scenarios. These hypothetical cases, developed from published HRV research and the authors’ applied experiences, are intended to help sport scientists and practitioners distinguish between positive physiological adaptations, acute homeostatic disturbances, and early signs of maladaptation. Although not specifically based on raw empirical datasets, the patterns shown above in Figure 2 and below in Table 5 reflect scientifically supported trends and offer practical guidance for interpreting HRV data within athlete monitoring systems.

It is important to recognize that the magnitude, timing, and interpretation of HRV responses may vary considerably between individuals. The onset and severity of such changes are influenced by several factors, including the athlete’s training background, baseline cardiorespiratory fitness, psychological stressors, nutritional status, and the duration or aggressiveness of a training cycle [105,106]. For example, more experienced or aerobically fit athletes may exhibit smaller day-to-day fluctuations in HRV metrics, even under intensified training loads, due to greater physiological resilience [107]. Therefore, individualized baseline tracking and contextual interpretation are essential for accurate HRV-based decision-making.

### 6.6. Practical Application and Calculation Schedule

To derive meaningful RMSSD_MEAN_ and RMSSD_CV_ values, HRV should be recorded daily by consistently following the HRV recording procedures detailed in Section 4 and outlined in Figure 1. The RMSSD_MEAN_ and RMSSD_CV_ should then be calculated from a completed 7-day block of daily RMSSD values. Each week’s set (e.g., Sunday → Saturday) is averaged to obtain the weekly mean (RMSSD_MEAN_), and the standard deviation from those same days is used to calculate the coefficient of variation (CV = SD/Mean × 100). This “fixed-week” approach enables clear week-to-week comparison and aligns with the evidence showing that weekly metrics best reflect chronic adaptation and acute recovery.

Alternatively, practitioners may calculate the mean and CV as rolling 7-day metrics, in which each new day updates the 7-day window. This approach supports HRV-guided training models, wherein each new RMSSD value is compared against the rolling average to inform real-time adjustments in training load or recovery strategies [108]. However, rolling seven-day values differ from the fixed weekly values (e.g., RMSSD_MEAN_ and RMSSD_CV_) discussed in this paper. Nevertheless, both approaches may be valuable, with fixed-week summaries used for monitoring long-term adaptation and weekly recovery status, while rolling metrics may be used for daily decision-making.

### 6.7. Section Summary

Based on the collective evidence, daily to near-daily HRV assessments, particularly using RMSSD, are recommended for optimizing the monitoring of recovery and training adaptations in athletes. This approach enables the calculation of both the mean and CV of HRV over the fixed weeks of a training program, with each serving distinct yet complementary roles. The weekly mean HRV reflects long-term autonomic trends and adaptations to training. On the other hand, the weekly CV of HRV identifies acute homeostatic disturbances, providing insight into recovery status and readiness to perform. Therefore, it is essential to calculate both metrics and interpret them separately, as they offer different insights into an athlete’s physiological state.

## 7. Additional Considerations

### 7.1. Automatic Calculation of Weekly Metrics

Despite the value of weekly HRV metrics, limited accessibility remains an issue. Many wearable devices do not measure HRV at all, and those that do often provide only a single daily HRV score without access to the underlying R–R interval data. There are a few platforms that automatically compute rolling seven-day RMSSD averages after a full week of data collection, which can be useful for short-term decision-making. This approach supports HRV-guided training models, wherein each new RMSSD value is compared against the rolling average to inform real-time adjustments in training load or recovery strategies [108]. However, rolling seven-day averages differ from the fixed weekly mean values (e.g., RMSSD_MEAN_) discussed in this paper, which are more appropriate for longitudinal monitoring (see Section 6, Data Interpretation). Both the weekly mean and CV must often be manually calculated from daily recordings, which may limit their accessibility for practitioners without data-analysis expertise or software.

### 7.2. Potential Limitations with Using RMSSD Alone

Although RMSSD selectively captures vagal activity [1], its capacity to reflect the full spectrum of autonomic balance, particularly in scenarios where sympathetic activation plays a central role, is limited [109,110,111]. In such cases, frequency-domain measures (e.g., LF, HF, LF/HF ratio) and non-linear methods (e.g., Poincaré plotting) may offer richer representations of both sympathetic and parasympathetic activity, as well as the adaptive complexity of cardiac control [4,112,113,114,115,116]. However, these approaches often require longer recordings and sophisticated analytic tools, which may be impractical in many applied sport settings [2,21,22].

Emerging research suggests that ratio-based, time-domain metrics may serve as practical surrogates for frequency-domain and non-linear methods. These approaches are proposed to complement RMSSD by providing a broader view of autonomic dynamics. For example, simplified indices such as SDNN/RMSSD or RMSSD/RR, as well as ultra-shortened time-domain modifications of the sympathetically mediated stress score, may provide additional insight into autonomic balance [42,111,117,118]. Such methods may be valuable when longer recordings or laboratory-based analyses are not feasible. However, further research is warranted to confirm their validity across different sports, training cycles, and populations before they are integrated into routine monitoring.

### 7.3. Integrating Subjective and Behavioral Indicators

Because HRV is an objective, physiological indicator, it may not fully reflect psychosocial stressors that influence training responses, such as insufficient or disrupted sleep, emotional distress, academic demands, or travel-related fatigue. These stressors can elevate sympathetic output without immediately suppressing parasympathetic activity and, hence, may result in little or no change in RMSSD despite increased allostatic load [88,119]. However, brief psychometric tools such as the Hooper Index [120,121], the Short Recovery and Stress Scale [122], the Total Quality Recovery Scale [123], the RESTQ-Sport [124], the Mclean questionnaire [125], the NASA Task Load Index [126], and the Pittsburgh Sleep Quality Index [126] have shown to be sensitive to both physical and psychological stress. When used alongside HRV, these measures can identify discrepancies between physiological signals and perceived recovery, offering early warning signs of non-functional overreaching or maladaptation [120]. Indeed, several studies have observed parallel changes in RMSSD and subjective recovery measures in athletes across training cycles [69,86,89,121]. Additional studies have supported the use of brief psychometric assessments as reliable indicators of training response, particularly when integrated with physiological monitoring [4,120].

### 7.4. Surrounding HRV Monitoring in Individual Athletes and Teams

Another consideration pertains to athlete compliance. As mentioned throughout the paper, daily HRV measures are important for monitoring athlete recovery and adaptation status. However, adherence to daily HRV monitoring is often high in the beginning, likely driven by novelty and motivation, but may decline over time unless intentional strategies are implemented to promote sustained compliance [85]. In team environments, promptly addressing any declines in daily recordings through communication with coaching staff can be especially valuable, as they play a pivotal role in reinforcing expectations, creating a culture of accountability, and connecting HRV tracking to tangible performance outcomes. For individual athletes, appropriately timed text message reminders and mobile-based cues can significantly improve adherence [127,128]. Practitioners may find it effective to text athletes proactively (e.g., first thing in the morning to prompt a recording) or reactively (e.g., after a missed day).

For both individuals and groups, athlete education is central to fostering long-term compliance. Athletes are more likely to integrate the practice into their daily routine when they understand the purpose of HRV tracking, such as its role in assessing recovery, identifying early signs of fatigue or illness, and guiding training load adjustments [129]. Personalizing the monitoring process by sharing meaningful feedback, such as how changes in HRV patterns relate to performance trends, further reinforces engagement by promoting a sense of ownership. Encouraging teams to view anonymized HRV trends collectively or recognizing athletes who demonstrate consistent engagement may not only educate the athletes about the importance of HRV monitoring, but could also build a shared culture.

Additionally, when working with large groups of athletes, cloud-based platforms that automatically update HRV data in real time are highly recommended for data management and compliance assurance. These systems streamline data management through secure, encrypted collection and monitoring of HRV in multiple athletes, regardless of location. Centralized access allows practitioners to identify trends and fluctuations related to athlete readiness, such as accumulated fatigue and the need for recovery interventions. Many platforms offer automated trend analysis, customizable dashboards, and integration with other health and performance data (e.g., GPS tracking, sleep monitoring, wellness questionnaires, etc.). When working with a group of athletes, practitioners and researchers are encouraged to consult the HRV device manufacturer to determine if their system supports cloud-based platforms or third-party data integration.

### 7.5. Signal Artifact Management in Mobile HRV Acquisition

When using mobile HRV devices, signal artifacts may arise from poor electrode or sensor contact, movement, ectopic beats, or transient vasoconstriction during recovery [60,130,131]. These disturbances distort beat-to-beat intervals and reduce device accuracy [131]. Standardized recording procedures, as described in Section 4, are therefore essential for data reliability [38]. Proper skin preparation can improve electrode or PPG contact, and many commercial platforms now incorporate automated artifact-detection and correction algorithms using threshold-based or adaptive filtering [132]. Although these methods enhance data quality, performance varies across devices and is rarely detailed in published research. Recordings with more than approximately 5% corrected beats should be discarded, as reliability declines substantially beyond this threshold [2].

Despite denoising strategies such as motion correction or filtering, HRV indices derived from exercise or immediate post-exercise recordings remain unstable [60,130]. Thus, daily HRV measurements should be performed under stationary, resting conditions to maximize accuracy and ensure meaningful assessment of recovery and adaptation.

### 7.6. Section Summary

While RMSSD remains a widely accepted HRV marker for monitoring athletes across training and competition periods, relying on it in isolation is not advised. At a minimum, RMSSD should be interpreted alongside simple psychometric variables, such as wellness questionnaires and training load indicators. Incorporating additional ultra-short HRV metrics, such as SDNN, to establish ratio-based parameters in conjunction with RMSSD shows promise for capturing sympathetic activity and autonomic balance, though additional validation is needed. Nevertheless, integrating multiple measures provides the most comprehensive approach to assessing training adaptation and recovery status in athletes.

## 8. Conclusions

HRV has become a popular “buzzword” in the realm of sport science. This review outlined the methodological considerations necessary for maximizing its utility in mobile settings, including the choice of metric and device, timing and posture of recordings, and data interpretation strategies. While a variety of HRV metrics exist, ultra-short (i.e., 1 min) RMSSD has emerged as the most user-friendly, practical, and robust for daily monitoring. Meaningful interpretation requires consistent daily measurements following standardized procedures. Weekly metrics are then derived from fixed 7-day blocks, with RMSSD_MEAN_ reflecting chronic adaptation, and RMSSD_CV_ representing short-term perturbations in homeostasis. This fixed week approach (e.g., Sunday-to-Saturday) allows reliable week-to-week comparisons across training periods.

Ultimately, integrating HRV monitoring into athlete management systems requires more than accurate data acquisition. It demands thoughtful implementation, consistent protocol adherence, and individualized interpretation. When HRV is combined with subjective wellness assessments and contextual training data, it evolves from a passive measurement into a dynamic decision-making tool capable of guiding recovery, optimizing training loads, and supporting long-term athletic development. As technology continues to advance and machine learning models become more sophisticated, the potential of HRV to deliver real-time, personalized insights will only expand. However, its value will remain contingent on informed application by practitioners who understand both its strengths and its limitations. With continued research, mobile HRV technologies hold strong potential to transform athlete monitoring and performance management systems.

## Figures and Tables

**Table 1:** Summary of literature search procedures for this narrative review.

Databases	Data Range Searched	Example Search Terms Used
PubMed Scopus Google Scholar	January–August 2024	“heart rate variability”, “HRV”, “RMSSD”, “ECG”, “time-domain”, “frequency-domain”, “electrocardiography”, “PPG”, “photoplethysmography”, “smartphone”, “ultra-short-term”, “athlete”, “athletic”, “training”, “adaptation”, “recovery”, “training load”, “readiness”, “fatigue”, “overreaching”, “overtraining”, “monitoring”, “performance”, “exercise”, “mobile”, “wearable”, “sports”, “coefficient of variation”, “weekly HRV”, “training adaptation”, “autonomic modulation”, “vagal”, “parasympathetic”, and device-specific terms (e.g., “Oura”, “Whoop”, “HRV4Training”, “FitBit”, “Polar”, “Apple”)

**Table 2:** Common heart rate variability metrics. Please note that this is not an exhaustive list. For a comprehensive review of available HRV indices, see Reference [2].

HRV Metric	Definition	Physiological Significance and Practical Implications
*Time-Domain Parameters Derived from Statistical Analyses*		
RMSSD	Root Mean Square of Successive R-R Differences	Parasympathetic marker; widely used and validated in athletes, especially in ultra-short-term mobile recordings, relatively easy to interpret
SDNN	Standard Deviation of Normal-to-Normal Intervals	Reflects overall autonomic activity; usable in ultra-short-term mobile recordings, though links to training status remain unclear
pNNx	Proportion (%) of R-R Intervals That Differ by >x milliseconds (e.g., >50 ms for pNN50, etc.)	Primarily parasympathetic; rarely used in athlete monitoring, with limited support for ultra-short-term use
SDNN/RMSSD	Ratio between SDNN and RMSSD	Sympathovagal balance; higher values suggest sympathetic shift. Emerging as a complementary athlete monitoring metric
*Frequency-Domain Parameters Derived from Power Spectral Density*		
HF Power	High frequency power (0.15–0.40 Hz)	Parasympathetic marker; typically requires ECG, specialized software, and ≥3 min lab recordings, often normalized as HFnu
LF Power	Low frequency power (0.04–0.15 Hz)	Controversial marker of mixed autonomic input; Requires ECG, specialized software, and ≥3 min recordings, often normalized as LFnu
LF/HF	Ratio between LF and HF	Sympathovagal balance, though debated; higher values = sympathetic shift; Requires ECG, specialized software, and ≥3 min recordings
*Non-linear Parameters Derived from Poincare Plotting*		
SD1	Short-term variability perpendicular to line of identity	Captures rapid parasympathetic changes; may require lab setup and specialized software, interpretation may be challenging
SD2	Long-term variability along the line of identity	Represents slower autonomic changes (both branches); lab-based, requires specialized software, and can be difficult to interpret
SD1/SD2	The ratio between SD1 and SD2	Purported marker of sympathovagal balance, though debated; may require lab setup, software, and can be difficult to interpret

**Table 3:** Overview of mobile HRV devices, providing example (Ex.) manufacturers, advantages and disadvantages regarding athlete monitoring, and summary of validity findings.

Type	Ex. Manufacturers	Key Advantages	Key Limitations	Validity Findings
Chest ECG Strap Monitor	Polar, Garmin, Movesense	High accuracy, ECG-like signal quality	May be inconvenient, accuracy influenced by sensor placement	High for ≥5 min recordings of time and frequency domains [16,60]. High for 1 min recordings of RMSSD [16]
Wrist-Worn PPG Device	Garmin, WHOOP, Apple, Fitbit, Empatica, Suunto	Continuous data, unobtrusive, additional health metrics	Sensor placement critical, can be costly	High for ≥5 min recordings of time and frequency domains [16,57,58,68]
Finger Ring PPG Device	Oura Ring	Continuous data, unobtrusive, suitable for nocturnal recordings	Sensor placement critical, can be costly, limited immediate feedback	High for ≥5 min recordings of RMSSD and HF [59,68]. Poor for LF and LF/HF [68]
Smartphone PPG App	HRV4Training, Elite HRV, ithlete, Pulse Sensor Pro	Low-cost, convenient, portable, simple, some only camera sensor	User-dependent accuracy, not continuous, cannot be used while sleeping	High for ≥5 min recordings of time and frequency domains [54]. High for 1 min recordings of RMSSD and SDNN [16,55]

ECG = electrocardiogram, PPG = pulse plethysmography, App = smartphone application.

**Table 4:** An example of daily RMSSD values across a week, as well as the weekly mean and CV calculated from the day-to-day values. Each day’s RMSSD represents a typical log-transformed and adjusted value.

Su	M	Tu	W	Th	Fr	Sa	MEAN	SD	CV
75	70	72	80	70	78	81	75.1	4.3	5.7%

Su = Sunday, M = Monday, Tu = Tuesday, W = Wednesday, Th = Thursday, F = Friday, Sa = Saturday, MEAN = RMSSD_MEAN_ calculated as the average of the 7-day period, SD = Standard deviation of the 7-day period, CV = RMSSD_CV_ calculated as SD/RMSSD_MEAN_ × 100.

**Table 5:** Practical Interpretation of RMSSD Mean (log-transformed and adjusted) and CV (%) in Athlete Monitoring. *Note: RMSSD values are hypothetical but based on published HRV patterns and experienced observations*.

Case	Training Context	Length (Weeks)	RMSSD_MEAN_	RMSSD_CV_	Interpretation	Practical Response	Key Supporting References
1	Acute increased training load	3	62 → 61	6.2 → 9.1	↑ CV, ↔ mean, homeostatic perturbation	Deload if ↑ CV persists or ↓ mean	[38,85,87,88,101]
2	Non-functional overreaching	8	60 → 45	4.8 → 11.8	↑ CV, ↓ mean; likely maladaptation	Deload, monitor closely for illness or burnout	[20,92,103,104]
3	Positive adaptation	24	55 → 72	7.1 → 4.5	↑ mean, ↓ CV; effective training	Maintain or gradually ↑ training load	[38,85,89,91,97,8]
4	Effective Taper	6	77 → 83	8.0 → 3.5	↑ mean with ↓ CV; strong readiness signal	Maintain taper; athlete likely primed to perform	[38,85,91,8]
5	Ineffective Taper	6	77 → 62	8.0 → 13.1	↓ mean with ↑ CV; indicates non-readiness	Deload; Emphasize recovery strategies	[71,85,95,99]
6	Onset of an acute illness	1–3	63 → 63	5.2 → 11.9	↑ CV with stable mean; immune system stress	Monitor for symptoms; Reduce load	[20,67,88]
7	Post-illness recovery	1–3	63 → 64	14.2 → 6.1	CV ↓; mean ↔ (or ↑); indicative of recovery	Gradually resume training; monitor closely	[20,67,88]
8	Active recovery microcycle	2–4	67 → 78	12.1 → 4.1	↑ mean, ↓ CV; signals readiness and recovery	Begin next training block	[38,85,95,8]
9	Psychological stress	1–3	71 → 70	2.9 → 10.1	Stress ↑ Sympathetic drive	Reduce stress; Psychometric monitoring	[88,105,106]
10	Overtraining Syndrome	15	81 → 50	3.3 → 9.0 → 3.0	CV ↑ and then ↓, mean ↓ from chronically ↑ TL	Deload; medical intervention	[20,103,104]

CV = Coefficient of variation, TL = training load, ↑ = increased or higher, ↓ = decreased or lower, ↔ = no change, → = indicates change from an earlier assessment to a later assessment.

## Data Availability

Due to the nature of this review, no new data were created.

## Abbreviations

The following abbreviations are used in this manuscript:

HRV	Heart Rate Variability
CV	Coefficient of Variation
R-R Interval	Time between successive R-waves in the QRS complex of an electrocardiogram
ECG	Electrocardiogram
RMSSD	Root Mean Square of Successive R-R Differences
SDNN	Standard Deviation of Normal-to-Normal Intervals
pNN50	Proportion of R-R Intervals That Differ by >50 ms
HF	High frequency power (0.15–0.40 Hz)
LF	Low frequency power (0.04–0.15 Hz)
SD1	Short-term variability perpendicular to line of identity of a Poincaré plot
SD2	Long-term variability along the line of identity of a Poincaré plot
PSD	Power Spectral Density
n.u.	Normalized Units
PPG	Pulse Plethysmography
CI	Confidence Interval
Ln	Natural Log-Transformed
FOR	Functional Overreaching
NFOR	Non-Functional Overreaching
SS	Stress Score
SPS	Sympathetic/Parasympathetic Ratio
MSS	Modified Stress Score
MSPS	Modified Sympathetic/Parasympathetic Ratio
SRSS	Short Recovery and Stress Scale
TQR	Total Quality Recovery Scale
RESTQ-Sport	Recovery-Stress Questionnaire
GPS	Global Positioning System
